# A Therapeutic Hepatitis B Virus DNA Vaccine Induces Specific Immune Responses in Mice and Non-Human Primates

**DOI:** 10.3390/vaccines9090969

**Published:** 2021-08-29

**Authors:** Dorien De Pooter, Ellen Van Gulck, Antony Chen, Claire F. Evans, Jean-Marc Neefs, Helen Horton, Daniel Boden

**Affiliations:** 1 Janssen Infectious Diseases, Janssen Research and Development, Division of Janssen Pharmaceutica NV, Turnhoutseweg 30, 2340 Beerse, Belgium; EVANGULC@its.jnj.com (E.V.G.); achen39@ITS.JNJ.com (A.C.); helen.horton@touchlight.com (H.H.); 2 Ichor Medical Systems Inc., 6310 Nancy Ridge Drive, Suite 107, San Diego, CA 92121, USA; cevans@ichorms.com; 3 Discovery Sciences, Janssen Research and Development, Division of Janssen Pharmaceutica NV, Turn-houtseweg 30, 2340 Beerse, Belgium; JNEEF1@its.jnj.com; 4 Janssen Infectious Diseases, Division of Janssen Pharmaceutica NV, 260 E. Grand Avenue, South San Francisco, CA 94080, USA; dboden@its.jnj.com

**Keywords:** therapeutic vaccination, hepatitis B surface antigen (HBsAg), hepatitis B virus (HBV) specific T-cells, HBV functional cure, non-human primate, electroporation

## Abstract

Despite the availability of an effective prophylactic vaccine for more than 30 years, nearly 300 million people worldwide are chronically infected with the hepatitis B virus (HBV), leading to 1 death every 30 s mainly from viral hepatitis-related cirrhosis and liver cancer. Chronic HBV patients exhibit weak, transient, or dysfunctional CD8^+^ T-cell responses to HBV, which contrasts with high CD8+ T-cell responses seen for resolvers of acute HBV infection. Therefore, a therapeutic DNA vaccine was designed, expressing both HBV core and polymerase proteins, and was sequence optimized to ensure high protein expression and secretion. Although the vaccine, administered intramuscularly via electroporation, had no effect on plasma viral parameters in a mouse model of persistent HBV infection, it did induce robust HBV-specific immune responses in healthy and adeno-associated hepatitis B virus (AAV-HBV) infected mice as well as in healthy non-human primates.

## 1. Introduction

Worldwide, approximately 300 million people are chronically infected with the hepatitis B virus (HBV) [1,2], even though a highly effective prophylactic vaccine against HBV has been available since 1982 [3]. When unvaccinated children get infected before the age of 18, 90% will develop chronicity [2] which ultimately results in liver damage in approximately 25% of cases [4]. This damage includes cirrhosis and hepatocellular carcinoma, which led to the death of an estimated 887,000 people in 2015 [2].

Current treatment for chronic hepatitis B (CHB) is based on life-long treatment with a nucleos(t)ide analogues (NA) to suppress HBV viral replication, or pegylated interferon (IFN) alpha, which is an immunomodulator that promotes antiviral immune response, for up to 1 year [5,6]. A sterilizing cure for HBV is thought to be unfeasible due to the presence of transcriptionally silent covalently closed circular DNA (cccDNA) in the nucleus of the hepatocytes. This cccDNA is not cleared with antivirals. Therefore, the goal of HBV treatment is functional cure, i.e., the sustained seroclearance of hepatitis B surface antigen (HBsAg) and undetectable HBV DNA in serum off treatment for ≥6 months, with or without HBsAg seroconversion into anti-HBs antibodies [7]. Currently, only 5% of the patients treated with NA showed HBsAg loss and are therefore eligible to stop treatment [5,6,8]. Due to the complexity of chronic HBV infection, it is generally accepted that a single therapy will not be sufficient to achieve functional cure when off treatment, which has spurred development of components for combination therapies. In this paper, we describe one aspect of a possible combination therapy, a highly immunogenic therapeutic DNA-based HBV vaccine. The vaccine discussed here could be part of a therapy that leads to an increase in the number of patients that no longer require therapeutic intervention.

Encouragingly, in >90% of adults who become infected with HBV, the infection is resolved, demonstrating that functional cure is achievable [9]. The patients who resolve their HBV infection have readily detectable ex vivo HBV-specific CD4^+^ and CD8^+^ T-cell responses, while such responses are less frequent and functionally impaired (exhausted) in chronic HBV-infected individuals [10]. These findings suggest that therapeutic vaccination should induce HBV-specific CD4^+^ and CD8^+^ T-cell responses that mimic those seen in resolvers. The aim of this vaccine is to induce a strong Th1 response to reverse the state of tolerance.

The therapeutic vaccine discussed here is a DNA vaccine that consists of a bacterial plasmid containing the gene(s) for HBV Core and Pol. The plasmid is delivered into the host cell by electroporation, which results in endogenous production, processing, and secretion of the encoded Core and Pol proteins. The antigens are then presented in the context of MHC class I and II molecules, which in turn leads to a cell-mediated immune response. The vaccine described here uses Core and Pol because they are both highly immunogenic and the coding sequences among different HBV genotypes are highly conserved. Core was selected to be part of the vaccine because the amount of Core specific T-cells in chronically infected patients is much lower than in patients who resolve their HBV infection [11]. It has also been shown that bone marrow transplants from donors with HBV Core-specific T-cells to individuals with chronic HBV infection resulted in resolution of HBV infection [12]. Additionally, the detection of HBV core and pol specific T-cells with in vitro proliferative ability is an indication that patients can be taken off NA treatment [13].

DNA-based vaccines are more attractive than viral-based vaccines because they can be used repeatedly to boost immune response without the development of anti-vector immune responses or significant safety concerns [14,15,16]. They have recently received increased attention due to evidence of efficacy for certain cancers in human clinical trials [17,18]. Delivery of DNA vaccines by electroporation has been shown to improve antigen expression and immunogenicity [19].

Herein we describe the design, optimization, and immunogenicity of a DNA vaccine comprising two non-infectious DNA plasmids (1:1 weight ratio): one encoding HBV core protein (Core) and the other encoding HBV polymerase (Pol).

## 2. Materials and Methods

### 2.1. Design of Core and Pol Amino Acid Sequences

The highly immunogenic HBV proteins Core and Pol were selected to be expressed using a DNA vaccine expression vector. The working hypothesis was that HBV antigen expression in vivo would result in increased frequencies of antigen-specific T-cells.

Globally, there are nine known HBV genotypes (A–I, with a possible new genotype ‘J’) that are unequally distributed over different geographic regions; the predominant genotypes in various regions are: A and D (Europe); D (Asia, and North Africa); C (Eastern and Southeastern Asia, Australasia, and Oceania); B (China, Southeast Asia, and Australia); D (Australasia and Oceania); E (western Sub-Saharan Africa); and F, G, and H in Latin America [20]. The heterogenicity of HBV sequences was considered in the vaccine design. The consensus sequence approach was chosen to generate an HBV vaccine sequence for regions with the highest prevalence of chronic HBV infection, i.e., Southeast Asia. The heterogeneity of HBV is relatively low due to the multiple overlapping reading frames in the HBV genome, limiting the emergence of resistance and other non-synonymous mutations. HBV Core and Pol amino acid (aa) sequences were aligned in silico using the CLUSTAL Omega tool (EMBL-EBI, Cambridge, UK) to generate a consensus sequence for HBV B, C, and D genotypes. In total, 110 HBV sequences were used for the alignment, including 34 genotype B, 43 genotype C, and 33 genotype D sequences. Once the consensus sequence of the Core protein was obtained, 34 amino acids of the highly positive-charged (arginine-rich) C-terminal RNA-binding domain of Core, including the nuclear localization signal [21], were deleted. The rationale was to limit any interference of HBV Core with human RNA and to facilitate protein secretion for subsequent cross-presentation. Similarly, using the consensus sequence of Pol protein, four aa mutations were introduced in the active sites of the polymerase and RNase H domains to inactivate the enzymatic activity of Pol.

### 2.2. Generation of Core/Pol DNA Vaccine Expression Plasmids

Efficient antigen expression is linked to a variety of vector features as well as the nucleotide sequence of the expressed protein. Codon adjustment of the latter to the codon usage of the species in which the antigen will be expressed can contribute to high protein expression. The consensus aa sequences of the C-terminally deleted Core and the inactivated Pol described in Section 2.1 were used to derive codon-optimized nucleotide sequences for HBV Core and Pol. These sequences, including appropriate restriction sites for subsequent cloning, were synthesized by GenScript (NJ, USA). Enhancer, regulatory elements, secretion signals, introns, resistance markers, and pUCori (origin of replication site) modifications were generated either by recombinational polymerase chain reaction or were made synthetically by Integrated DNA Technologies (IA, USA). DNA constructs were generated by standard molecular cloning methods applying forced cloning via restriction enzymes followed by transformation into chemically competent Top10 cells (Thermo Fisher Scientific, MA, USA). The following expression vectors were used: the commercially available expression plasmids pcDNA3.1 and pVax1 (Thermo Fisher Scientific, MA, USA), and the in-house engineered constructs pVD, pVK, pDK, and pDF described in Table 1. Any new molecular DNA entity was sequence-verified and plasmid DNA subsequently upscaled for downstream cellular expression experiments using the Plasmid Plus Midi kit (Qiagen, Hilden, Germany). Table 1 summarizes all plasmids evaluated.

### 2.3. HBV Core and Pol Expression Strategies

The choice of the Pol 2 promoter driving the transcription of the transgene coding sequences is important, such as the strong immediate early cytomegalovirus (CMV-IE) promoter that can be used in DNA vaccine vectors. Furthermore, RNA sequences that stabilize the expressed transcript, enhance its nuclear export, and improve transcriptional-translational coupling are known to lead to increased protein expression. The inclusion of a secretion signal peptide is applied in certain DNA vaccine approaches, resulting in more robust antigen expression and cross-presentation in antigen-presenting cells (APCs). The overall plasmid size will influence transfection (electroporation) efficiency, as it decreases significantly with larger plasmids. The orientation of the bacterial replication origin and antibiotic resistance cassette in respect to the antigen expression cassette may affect protein expression in mammalian cells as well as the sequence composition of the antibiotic gene that is normally adjusted to the *E. coli* codon usage. Three different molecular approaches were evaluated to obtain maximum and similar expression of both Core and Pol proteins.

The first vector design was based on fusing the HBV Core and Pol protein in-frame, resulting in a single large Core-Pol fusion protein (pcDNA-CP, Table 1). A small AGAG spacer was inserted between both proteins to avoid homology to a host protein (dopamine receptor) created by direct fusion of both HBV proteins. Correct folding of either individual HBV protein would likely be abrogated due to the attached protein cargo on the C-terminus of Core and the N-terminus of Pol. The lack of protein folding is not a concern for vaccines devised to drive T-cell responses.

The second strategy was to express both antigens from one plasmid by means of a ribosomal slippage site, also called cis-hydrolase site, between the Core and the Pol coding sequences (pcDNA-CFAP, Table 1). The result was a bicistronic expression vector expressing individual Core and Pol proteins from a single mRNA. The F2A slippage site from the foot-and-mouth disease virus (FMDV) was chosen to be inserted between the Core and Pol proteins. The Core protein contains, upon ribosomal slippage, a 21aa C-terminal tail, and the Pol protein contains one N-terminal proline residue addition.

The third Core-Pol expression strategy comprised two separate plasmids encoding for HBV Core and Pol antigens, respectively. The native configuration of each protein was maintained without addition of any heterogenous sequence. However, the in vivo application required either a mixture of both plasmids delivered in one anatomical site or two separate immunizations with each single expression plasmid.

### 2.4. Transfection and Protein Extraction

HEK293T cells (ATCC, Manassas, VA, USA) were seeded in 6-well plates in antibiotic-free medium (Dulbecco’s modified Eagle medium; Thermo Fisher Scientific Europe bv) with 10% heat-inactivated fetal bovine serum (Biowest, Nuaillé, France) and 1% L-glutamine (Thermo Fisher Scientific Europe bv). A day later, cells were transfected with DNA plasmids using polyethylenimine (PEI; Polysciences, Badener, Germany) (PEI/DNA 6/1 ratio; 500 µL/well). After overnight incubation (37 °C, 5% CO_2_), medium was replaced with the same medium containing an antibiotic (0.04% Gentamicin; Thermo Fisher Scientific Europe bv). Forty-eight hours after transfection, supernatant and/or lysates were collected, and proteins were extracted using the M-PER protein extraction kit (Thermo Fisher Scientific Europe bv) according to the manufacturer’s instructions.

### 2.5. Western Blotting

The total protein concentration was determined using Pierce BCA protein assay kit (Thermo Fisher Scientific Europe bv) following the manufacturer’s instructions. Western blotting was performed using a 4–12% Bis-Tris protein gel, with MES SDS running buffer and transfer onto a polyvinylidene difluoride membrane (respectively NP0322Box, NP0002, and IB4010-02, Thermo Fisher Scientific Europe bv). For Western blotting, a 10 µg/sample was used for lysates and a fixed amount of supernatant. Proteins were visualized using antibodies against Core (Dako, Glostrup, Denmark) and Pol (Santa Cruz Biotechnology, Dallas, TX, USA); goat anti-rabbit IgG horseradish peroxidase (GE Healthcare Life Sciences, Hoegaarden, Belgium) and goat anti-mouse IgG (H + L) horseradish peroxidase (Thermo Fisher Scientific Europe bv) secondary detection systems were employed. Read out was performed using SuperSignal West Femto substrate (Thermo Fisher Scientific Europe bv).

### 2.6. Plasmid Preparation and In Vivo Studies

Plasmids were prepared by Aldevron (Freiburg, Germany) for in vivo mouse studies and by GeneWiz (Leipzig, Germany) for non-human primate studies. Plasmids were formulated in sterile phosphate buffered saline (PBS) and mixed at equal volume.

BALB/c mouse studies were performed at Ichor Medical Systems (San Diego, CA, USA), while non-human primate studies were performed at Charles River Laboratories (Montréal, Canada) and used the plasmids with the pDK backbone. For mouse studies performed in-house with C57BL/6 mice, the Core and Pol plasmids were in a pDF vector, i.e., the same backbone as pDK but with the kanamycin-resistance cassette replaced with an ampicillin-resistance cassette (Table 1). The control vaccine used for in vivo studies was always in the pDK backbone, without Core or Pol.

### 2.7. Immunization of Healthy Mice

Female BALB/c mice (8–10 weeks old; Envigo, Indianapolis, IN) were obtained by Ichor Medical Systems (San Diego, CA, USA). In-house experiments were performed with male C57BL/6 mice (6–8 weeks old; Janvier Labs, Le Genest-Saint-Isle, France).

To improve the immunogenicity of the vaccine, the plasmids were administered intramuscularly via electroporation [22,23,24]. BALB/c mice or C57BL/6 mice were electroporated (TriGrid electroporation system, Ichor Medical Systems, San Diego) with the DNA plasmids on days 0 and 14 for assessment of fusion plasmids. For the immune interference and dose dilution experiments, vaccinations were performed on days 0 and 21. The plasmids were dissolved in PBS and administered intramuscularly in the cranialis tibialis (20 µL) via electroporation. Plasmid dosing varied between experiments from 0.2 up to 20 µg of total DNA. Samples were harvested 7 days after the last vaccination.

Mice were anesthetized (isoflurane via inhalation, 4% induction, and 2% maintenance) for electroporation and euthanized at the end of the experiment by CO2 inhalation.

### 2.8. Monitoring of Induced T-Cell Responses

The IFN-γ enzyme-linked immune absorbent spot (ELISpot) assay was performed on splenocytes or intrahepatic lymphocytes (IHLs) from BALB/c and/or C57BL/6 mice. Spleens were isolated from mice; tissue disruption was performed using a GentleMACS dissociater (Miltenyi Biotec, Bergisch Gladbach, Germany) or by pressing through a 100 µm nylon cell strainer (Falcon; Corning, NY), and cells were added to coated ELISpot plates (Mabtech, Nacka Strand, Sweden). IHLs were obtained by perfusing the liver with PBS; tissue disruption was performed using a GentleMACS dissociater, followed by a 33.75% (*v*/*v*) Percoll^®^/PBS/2% fetal calf serum gradient. Hepatocytes were separated from lymphocytes by centrifugation at 700× *g* (12 min, room temperature, maximum acceleration, brake at 1). The IHLs were aspirated carefully and tested using IFN-γ ELISpot. Splenocytes were tested at 200,000 cells/well and IHLs were tested at 100,000 cells/well on IFN-γ ELISpot plates.

All cells were stimulated with overlapping peptide pools covering the entire Core and Pol sequences (JPT, Gladback, Germany) on pre-coated ELISpot plates (Mabtech, Nacka strand, Sweden) to measure the amount of cells that secrete IFN-γ. As a negative control, cells were stimulated with dimethyl sulfoxide (DMSO). After overnight stimulation, plates were developed following the manufacturers’ instructions (Mabtech, Sweden). The amount of spot forming cells (SFC) was counted using an ELISpot reader (CTL, Cleveland, OH, USA). The DMSO (mock control) background was subtracted from all responses. Immunogenicity results are presented as the number of SFC per million of PBMC. Results are shown as means ± standard deviation. Due to the large size of the Pol protein, the peptide pool for this protein was split into two peptide pools, Pol1 and Pol2. The final result was expressed as the total Pol response (which was the sum of Pol1 and Pol2 per responses per animal).

### 2.9. Measuring Viral Parameters in the AAV-HBV Infection Mouse Model

Because HBV does not infect mice, a model of HBV infection with a recombinant adeno-associated virus (rAAV) was used to deliver the repeat area of the HBV genome to mice, resulting in long-term production of HBV virions by liver cells, absence of HBV-specific immune responses, and high levels of HBV viremia [25]. C57BL/6 mice were infected with rAAV8-1.3HBV (Fiveplus Medical Institute, Shanghai, China) via the tail vein at a multiplicity of infection of 10^11^ viral genome equivalent (vge) per mouse. Vaccination was performed when persistent HBV viremia was reached (day 28 after infection) and 21 days later [25,26], via electroporation with either vaccine (20 µg, n = 8) or control (backbone only; 20 µg; n = 8). Seven days after the last immunization, splenocytes and IHLs were harvested and assessed for HBV specific T-cell responses using IFN-γ ELISpot as described above. Blood (150 µL) for viral parameters was collected via saphenous vein at days 0, 7, 14, 21, and 28 post-vaccination, and serum was prepared and stored at −80 °C until assayed. The HBV viral load, HBsAg, and hepatitis B e-antigen (HBeAg) levels (diluted 1/100 in serum) were evaluated as described previously [27].

### 2.10. Non-Human Primate Study (Charles River, Senneville, Canada)

Male cynomolgus macaques, between 2 and 4 years old with a weight varying from 2.8–3.8 kg (Covance Research Products Inc, Texas, TX, USA), were vaccinated (2 mg; n = 5/group) on days 0, 36, and 62. Prior to each dosing, the monkeys were anesthetized with ketamine/acepromazine cocktail (100 mg/mL and 10 mg/mL), respectively, at 0.14 mL/kg in the biceps. HBV vaccine was administered by IM injection followed by EP (TriGrid) into the lateral compartments of the quadriceps (vastus lateralis).

Blood samples were collected on day –1 (pre-bleed) and on day 76. Peripheral blood mononuclear cells (PBMCs) were isolated using a Ficoll-Paque density gradient and plated at 200,000 cells/well to assess T-cell responses with IFN-γ ELISpot against DMSO (mock control), HBV Core, Pol1, and Pol2 peptide pools (2 µg/mL). ELISpot was developed and presented as described in Section 2.8.

### 2.11. Statistical Analyses

Statistical testing between groups was performed using two-way ANOVA test on log_10_ transformed data. A significant difference between two groups was reached when the *p*-value was ≤0.05.

### 2.12. Ethics Statement

In vivo experiments were performed within guidelines established by the Janssen Pharmaceutica N.V. Institutional Animal Care and Use Committee. The local Johnson and Johnson Ethical Committee approved all experimental protocols performed at Janssen. Experiments were performed following the guidelines of the European Community Council directive of 24 November 1986 (Declaration of Helsinki 86/609/EEC). The criteria of the American Chemical Society ethical guidelines for the publication of research were met. Every effort was made to minimize animal discomfort and to limit the number of animals used.

Studies at Ichor were performed under protocols approved by Ichor Medical Systems’ Institutional Animal Care and Use Committee and following the guidelines of the Guide for the Care and Use of Laboratory Animals published by the National Academy of Sciences (US). Every effort was made to minimize animal discomfort and to limit the number of animals used.

Non-human primate studies at CRL were performed using the Association for Assessment and Accreditation of Laboratory Animal Care guidelines.

## 3. Results

### 3.1. Maximizing Protein Expression and Secretion

**Figure 1:**
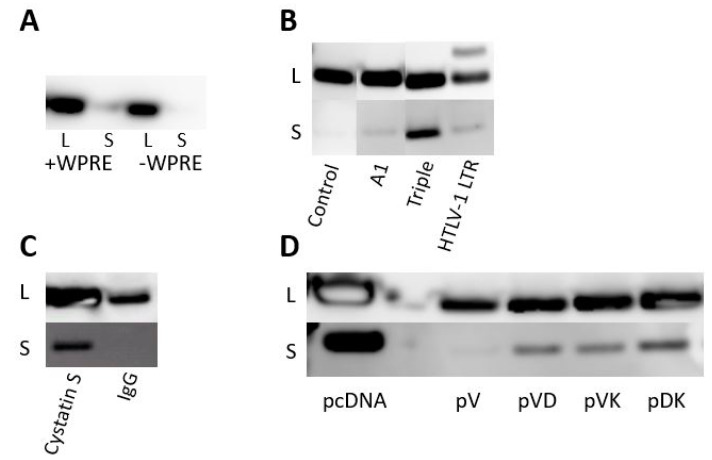
Western blot detection of Core protein in lysate (L) and supernatant (S) of HEK293T cells transfected with plasmids containing different enhancer elements, signal peptides, and backbones. (**A**) With and without enhancer element Woodchuck Posttranscriptional Regulatory Element (WPRE) (complete figure in Figure S1A). (**B**) Comparison between A1 intron, triple enhancer, and HTLV-1 R sequence (complete figures in Figure S1B,C). (**C**) Comparison between Cystatin S and IgG heavy chain signal (complete figures in Figure S1D,E). (**D**) Comparison between control backbone pcDNA3.1, pVax1-Core (pV), pVax1-Core without antibiotics (pVD), pVax1-Core with reverse orientation of Ori-kanamycin cassette (pVK), and pVax1-Core with pUCori-Amp_prom_-KanR cassette (pDK) (complete figures in Figure S1F,G).

Four different elements were evaluated to enhance protein expression via stabilizing the primary transcript, facilitating its nuclear export, and/or improving transcriptional-translational coupling. The first element tested was the Woodchuck HBV Post-transcriptional Regulatory Element (WPRE) (pcDNA-WPRE-Core, Table 1), which has been shown to enhance luciferase and green fluorescent protein reporter expression up to 8-fold [28]. When protein expression was assessed by Western blotting following transfection of the constructs into HEK293T cells, the WPRE inclusion only slightly increased cellular expression over parental Core plasmid, whereas it had no effect on secreted Core protein yield (Figure 1A).

Three additional expression-boosting strategies were evaluated: (1) inclusion of an intron/exon sequence derived from the human apolipoprotein A1 precursor (pcDNA-A1-Core, Table 1); (2) inclusion of the untranslated R-U5 domain of the human T-cell leukemia virus type 1 (HTLV-1) long terminal repeat (LTR) (pcDNA-HTLV-1-Core, Table 1), which was shown to boost protein expression up to 2-fold when located downstream of a strong constitutive promoter [29]; and (3) inclusion of a triple enhancer composed of three consecutive elements: the HTLV-1 LTR mentioned above, a synthetic rabbit β-globin intron, and a splicing enhancer [30] derived from expression plasmid NTC8385 (Nature Technology, Lincoln, NE) (pcDNA-NTC-Core, Table 1). These three different enhancer modules were introduced between the CMV promoter and Core gene. When Core protein expression from the different constructs transfected into HEK293T cells was analyzed by Western blotting and compared with the parental Core plasmid, the greatest intracellular and extracellular levels were observed with the triple enhancer construct (Figure 1B).

The addition of an N-terminal secretion signal peptide to any given antigen will direct the protein into the cellular secretory pathway [31]. For vaccine purposes, antigen secretion is much desired for the uptake and processing of antigens by APCs. Peptides derived from the processed antigens will be presented by either MHC class I or class II molecules on the cell surface of the APCs accessible for antigen recognition by CD4^+^ and CD8^+^ cells. In addition to this mechanism, called cross presentation, the inclusion of a signal peptide also improves cytosolic antigen expression. This improvement is especially true for proteins that are naturally targeted predominantly to the nucleus, as are HBV Core and Pol.

Different signal peptides introduced in frame at the N-terminus of HBV Core were evaluated: the Ig heavy chain gamma signal peptide SPIgG (pcDNA-HC-Core, Table 1) and the cystatin S precursor signal peptide SPCS (pcDNA-CystS-Core, Table 1). Signal peptide cleavage sites were optimized in silico for Core fusion using the Signal P prediction program (Supplementary Table S1). Secretion efficiency was determined by analyzing Core levels in the supernatant of HEK293T cells transfected with plasmids encoding Core with the signal peptides. The cystatin S signal peptide clearly outperformed the IgG signal peptide, leading to substantial Core secretion into the supernatant (Figure 1C).

Thus, the triple transcriptional enhancer element and the cystatin S secretion signal were implemented in lead Core and Pol expression plasmid design.

### 3.2. DNA Vaccine Vector Optimization

Initial in vitro expression studies were conducted with the widely used expression plasmid pcDNA3.1, which might not be suitable for use in human studies due to its ampicillin and neomycin resistance markers and the inclusion of the SV40 origin of replication. Hence, the HBV Core coding sequence was transferred into the commercially available DNA vaccine vector pVax1 (Table 1), which has been widely used in clinical studies and is, thus, generally accepted by regulatory authorities. The expression cassette in pVax1 consists of a human CMV-IE promoter driving the gene of interest terminated by the bovine growth hormone-derived polyadenylation sequence (pA). The pUCori replicon and a kanamycin resistance (KanR) gene driven by a small prokaryotic promoter allow for bacterial plasmid propagation.

When the HBV Core expression cassette sequence was transferred from pcDNA3.1 into the pVax1 backbone (pVax1-Core, Table 1), a marked reduction of protein expression was noticed compared to the yield obtained with the original pcDNA3.1 backbone (Figure 1D). Low expression in the pVax1 vector has been previously reported [32] and is associated, at least partially, with the KanR marker that was shown to act as a potential transcriptional silencer on the eukaryotic promoter [33]. The basic elements in pVax1 that differ from pcDNA3.1 are the KanR gene combined with its native promoter and the orientation of the replicon, which is counterclockwise in pcDNA3.1.

Several variants of pVax1-Core were made to enhance protein expression. To explore the hypothesis that the antibiotic KanR cassette may inhibit protein expression in pVax1-Core, the antibiotic-free plasmid pVD-Core (Table 1) was engineered. In this construct, the complete kanamycin gene expression cassette was replaced with the ccdA/ccdB type II toxin-antitoxin system [34]. The lack of antibiotics could complicate the upscaling process of the plasmid, although this approach completely removes the risk of any hypersensitivity towards kanamycin. To address whether the orientation of the origin of replication and/or antibiotic cassette could inhibit Core expression, the variant pVK-Core (Table 1) was engineered by reversing the orientation of the complete pUCori-KanR cassette. The third Core plasmid variant pDK-Core (Table 1) was generated to rule out any suppressive activity on protein expression through the kanamycin promoter or kanamycin coding sequence. Therefore, the KanR expression cassette in pVK-Core was replaced by the ampicillin promoter followed by a codon-optimized KanR gene (pUCori-Amp_prom_-KanR_cod_). Core expression from these different Core pDNA variants was assessed by Western blotting after transfection into HEK293T cells (Figure 1D). The most robust Core expression was seen in the cell lysate and supernatant for pDK-Core, followed by pVK-Core, pVD-Core, and pVax1-Core. Hence, the pDK plasmid was selected as the lead vaccine vector.

### 3.3. Assessment of Fusion Plasmids

**Figure 2:**
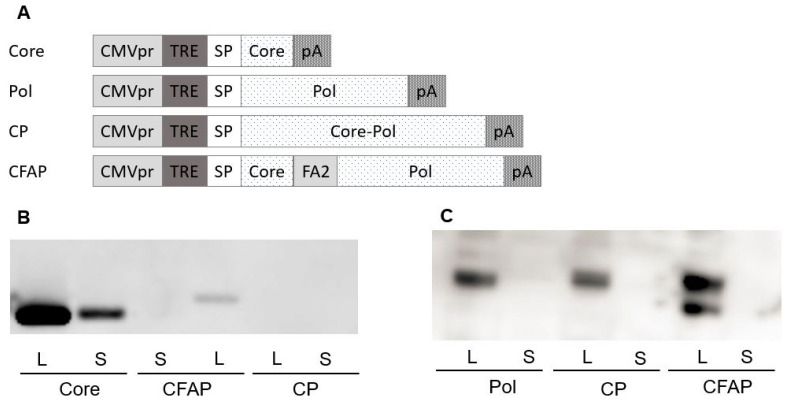
Comparison of Core and Pol expression from different DNA plasmids encoding Core and/or Pol. (**A**) Schematic overview of the different plasmids containing Core and/or Pol evaluated for expression in vitro and in vivo; all plasmids include the CMV promotor, TRE (Triple RNA Enhancer), and signal peptide (SP) upstream of the coding region, which is flanked on the 3′ end by a polyA tail. (**B**) Western blot detection of Core protein read out for lysate (L) and supernatant (S) of HEK293T cells transfected with different plasmids shown in Figure 2A (complete figure in Figure S2A). (**C**) Western blot detection of Pol protein in lysate (L) and supernatant (S) of HEK293T cells transfected with different plasmids shown in Figure 2A (complete figure in Figure S2B).

Four different HBV *Core/Pol* expression cassettes (Figure 2A) were introduced into pDK, resulting in the following constructs: two individual Core and Pol expression vectors (pDK-Core and pDK-Pol, Table 1); a Core-Pol fusion vector (pDK-CP, Table 1) with an intergenic AGAG spacer to avoid homology to host proteins created by the direct fusion of both HBV proteins; and the fusion construct pDK-CFAP (Table 1), whereby Core and Pol proteins were separated by a ribosomal F2A slippage site FMDV [35], resulting in the formation of two individual proteins from a single mRNA transcript.

Protein expression from these four plasmids was first evaluated after transfection in HEK293T cells by Western blotting using both Core (Figure 2B) and Pol (Figure 2C) specific antibodies. The findings show that the Core expression was much higher when Core is expressed from a non-fusion plasmid compared to the Core expression observed for the CFAP fusion. There was no Core expression detected for the CP fusion plasmid. A possible explanation was that since CP fusion results in one large Core-Pol protein, the configuration and folding of the protein inhibited the binding of the Core antibody. This explanation was confirmed in an additional Western blot showing that the Pol protein runs slightly higher for CP fusion compared to Pol protein alone (data not shown).

Core protein could only be detected in the transfection culture supernatants of the non-fused Core plasmid. For the CP fusion, this finding might also be due to configuration and folding issues; for the CFAP fusion, it appeared that Core was not secreted.

Pol expression was observed in the cell lysates for all transfections (Figure 2C). Expression with the Pol plasmid or the CP fusion plasmid was comparable, whereas the Western blot showed two different bands for the CFAP fusion (Figure 2C). The lower band was Pol protein, and the upper band was likely the Pol protein with Core and/or 21aa tail attached to it. Pol was not secreted in the supernatant for any of the transfected plasmids, which was expected since nuclear proteins such as DNA polymerases are difficult to engineer for secretion.

Based on these data, Core expression was optimal for Core plasmid alone compared with the fusion plasmids. For Pol expression, results were comparable between the fusion and the separate Pol plasmids. To further differentiate between the different constructs, immunogenicity was tested in BALB/c mice.

### 3.4. In Vivo Immunogenicity of Core and Pol Plasmids Compared with Fusion Plasmids

**Figure 3:**
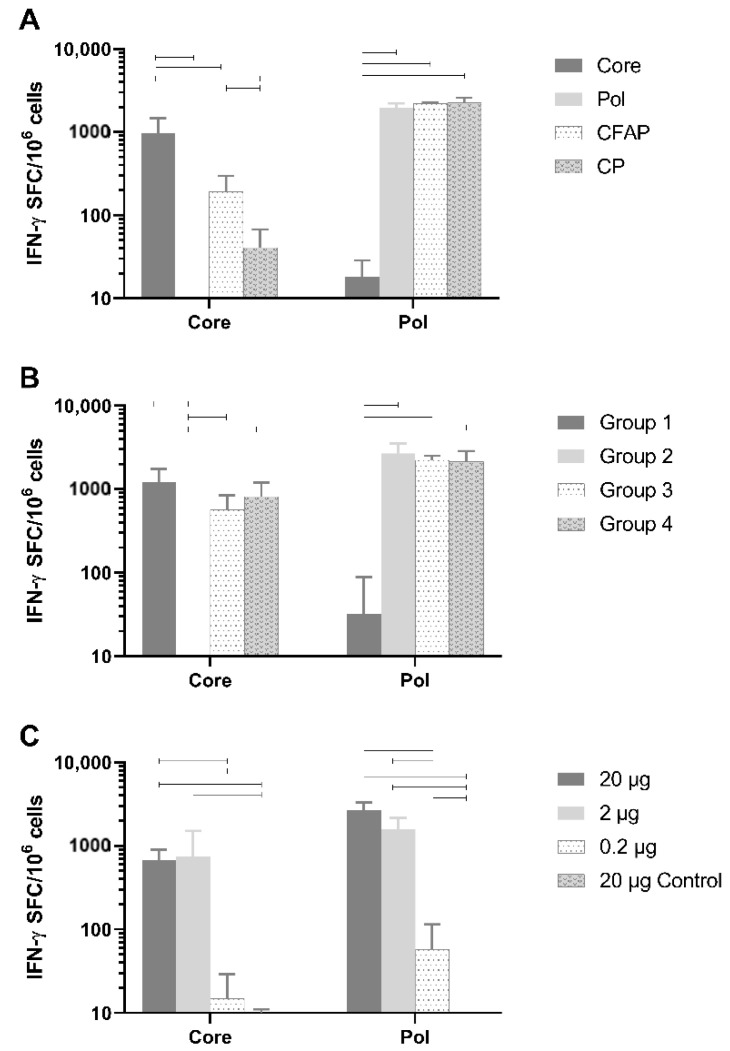
Immunogenicity of Core and Pol fusion plasmids, immune interference evaluation of Core and Pol, and dose finding study of DNA vaccine in healthy BALB/c mice. (**A**) Core and Pol specific T-cells (number of IFN-γ SFC/million cells) in healthy BALB/c mice (n = 6) after IM electroporation delivery of pDK-Core or pDK-Pol plasmids versus fusion plasmids pDK-CFAP and pDK-CP. (**B**) Core and Pol specific T-cell responses (number of IFN-γ SFC/million cells) in healthy BALB/c mice (n = 6 per group) after IM electroporation delivery of pDK-Core or pDK-Pol plasmids alone (Group 1 and 2; 5 µg per injection site, bilateral) compared to a mixture of pDK-Core and pDK-Pol plasmids (Group 3; 5 µg per plasmid per injection site, bilateral) or compared to injecting pDK-Core plasmid in the left cranialis tibialis and pDK-Pol plasmid in the right cranialis tibialis (Group 4; 10 µg per plasmid). (**C**) Core and Pol specific T-cell responses (number of IFN-γ SFC/million cells) in healthy BALB/c mice (n = 8) after IM electroporation delivery of 1:1 mixture of pDK-Core and pDK-Pol plasmid at different doses (total DNA: 20 µg; 2 µg and 0.2 µg) versus IM electroporation delivery of 20 µg of control plasmid (backbone without Core or Pol). Dosing and sampling performed as described in Materials and Methods. Statistical significance (*p* ≤ 0.05) is indicated above the bars.

Healthy naive BALB/c mice were vaccinated with 20 µg of DNA plasmid (n = 6/group) using the TriGrid electroporation device intramuscularly. Robust T-cell responses against Core peptides by splenocytes were detected (Figure 3A) and were specific to Core since no Core response was seen after vaccination with the Pol plasmid (mean: 2.1 IFN-γ spot-forming cells [SFC]/million cells; *p* < 0.0001) or the empty plasmid (Figure 3A). Responses to non-fusion Pol (1959.6 SFC/million cells) were also specific since no Pol response was measured after vaccination with Core plasmid (18.3 IFN-γ SFC/million cells; *p* < 0.0001) or the empty plasmid (data not shown).

The Pol responses between plasmids were comparable (non-fusion Pol versus CP and CFAP: *p* = 0.955 and *p* = 0.975, respectively; CFAP versus CP *p* = 0.9997). Fusion constructs, CFAP and CP, gave a significantly reduced Core response (192.9 and 40.8 IFN-γ SFC/million cells, respectively) compared to the non-fusion Core construct (972 IFN-γ SFC/million cells; *p* < 0.0001).

Evaluation of both in vitro and in vivo results demonstrated that the non-fusion plasmid gave the highest protein expression and antigen-specific T-cell responses following immunization, especially for Core.

### 3.5. Assessment of Immune Interference

As noted above, when Core was combined in a fusion construct with Pol, its immunogenicity was significantly reduced (Figure 3A). The reason for this reduction could be due to the fusion plasmid itself, or to immune interference between Core and Pol. In the latter case, one antigen will compete with the other, resulting in reduced responsiveness against one of the antigens [36]. Therefore, an experiment was performed in healthy BALB/c mice to determine if Core and Pol could be administered together without impacting antigen immunogenicity (Figure 3B; n = 6/group). As a control group, the pDK-Core and pDK-Pol were administered alone (Group 1 and Group 2, respectively). For Group 1, there was, as expected, a high response to Core (1226.3 IFN-γ SFC/million cells). In this group, there was an anomalous response (137 IFN-γ SFC/million cells) to the Pol2 peptide pool. In Group 2, where pDK-Pol was administered alone, no response for Core was measured (<50 IFN-γ SFC/million cells) compared to a high response for Pol (2688.3 IFN-γ SFC/million cells).

There was no significant difference after administering a mixture of Core and Pol plasmids in the same injection site (Group 3: Core 570.8 IFN-γ SFC/million cells; Pol 2233.3 IFN-γ SFC/million cells) compared to administering Core and Pol to different legs (Group 4: Core 819.2 IFN-γ SFC/million cells, *p* = 0.732; Pol 2148.8 IFN-γ SFC/million cells, *p* = 0.996). Additionally, no differences were seen when comparing the combination therapies of Group 3 and Group 4 with the single plasmid group (Group 1 and Group 2). This was the case for both Core (*p* = 0.152 for Group 1 versus Group 3, *p* = 0.672 for Group 1 versus Group 4) and Pol (*p* = 0.974 for Group 2 versus Group 3, *p* = 0.918 for Group 2 versus Group 4).

Collectively, these data show no evidence of immune interference between the Core and Pol antigens when plasmids were co-administered at the same site.

### 3.6. Defining the Optimal Dose of the Therapeutic Vaccine in BALB/c Mice

A dose-range finding study was performed by immunizing healthy BALB/c mice with a 1:1 mixture of pDK-Core and pDK-Pol plasmids. The vaccine was dosed intramuscularly at 20 µg, 2 µg, or 0.2 µg (n = 8/group) using the TriGrid electroporation device. As a negative control, a group of animals received empty pDK plasmid with electroporation (20 µg).

A dose-dependent increase in immunogenicity was observed for both the Core and Pol-specific IFN-γ ELISpot responses (Figure 3C). The vaccine generated high IFN-γ ELISpot responses even in mice receiving only 2 µg of total DNA. Responses were significantly higher for the 20 µg (Core 670.3 IFN-γ SFC/million cells; Pol 2685.9 IFN-γ SFC/million cells) and 2 µg (Core 741.9 IFN-γ SFC/million cells; Pol 1600.3 IFN-γ SFC/million cells) doses compared to the 0.2 µg dose (Core 15 IFN-γ SFC/million cells; Pol 58.1 IFN-γ SFC/million cells) for both Core (*p* < 0.0001) and Pol (*p* < 0.0001).

As the antiviral effects of the vaccine were assessed in AAV-HBV infected C57BL/6 mice (see below), the immunogenicity of the vaccine was also determined in healthy C57BL/6 mice dosed with 20 µg of the vaccine (n = 8/group). The magnitude of responses was comparable to that measured in BALB/c mice (Figure S3).

### 3.7. Efficacy of the Vaccine in AAV-HBV Infected C57BL/6 Model on Viral Parameters

**Figure 4:**
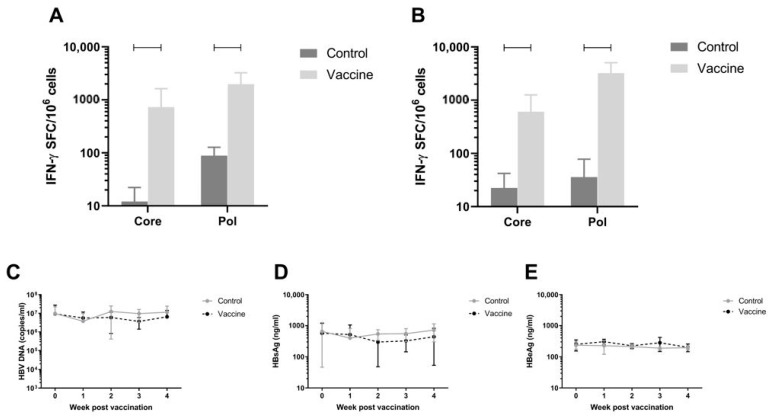
Immunogenicity and effect on viral parameters of vaccination with pDK-Core and pDK-Pol in AAV-HBV infected C57BL/6 mice. Core and Pol specific T-cell responses at day 56 after rAAV-HBV infection in (**A**) spleen and (**B**) intrahepatic lymphocytes after vaccination with pDK-Core and pDK-Pol or control (backbone only) plasmids at day 28 and 49 after infection (n = 8). Viral parameters (**C**) total HBV DNA (copies/mL), (**D**) HBsAg (ng/mL), and (**E**) HBeAg (ng/mL) were measured in the blood during the experiment. Infection, dosing, and sampling were performed as described in Materials and Methods. Statistical significance (*p* ≤ 0.05) is indicated above the bars.

To assess antiviral efficacy of the vaccine, the AAV-HBV infection mouse model was used. C57BL/6 Mice were infected with rAAV-HBV8-1.3HBV via the tail vein (10^11^ viral particles as described in Materials and Methods). When persistent HBV viremia was reached (day 28), mice were immunized twice with a 1:1 mixture of pDK-Core and pDK-Pol plasmids via electroporation (n = 8/group). HBV-specific T-cell responses were measured on day 56 post infection by IFN-γ ELISpot in splenocytes and IHLs (Figure 4A,B respectively). There was a significant increase in Pol specific T-cell response in the 20 µg dose compared to the negative control pDK backbone in splenocytes (730.3 IFN-γ SFC/million cells versus 12.1 IFN-γ SFC/million cells; *p* < 0.0001) and IHL (606.6 IFN-γ SFC/million cells versus 22.5 IFN-γ SFC/million cells; *p* < 0.0001). A similar result was seen for Core-specific T-cell responses, with significantly higher responses in vaccinated mice (spleen 730.3 IFN-γ SFC/million cells; IHL 606.6 IFN-γ SFC/million cells) compared to animals that were dosed with control (spleen 12.1 IFN-γ SFC/million cells, *p* < 0.0001; IHL 22.5 IFN-γ SFC/million cells, *p* = 0.0005).

Despite the robust Core and Pol specific T-cell responses induced by the immunization, there was no effect on plasma viral parameters in the mice (Figure 4C–E).

### 3.8. Immunogenicity in Non-Human Primates

Next, immune responses were evaluated in a larger species. Immunogenicity after three doses of the mixture of pDK-Core and pDK-Pol plasmids was evaluated in healthy cynomolgus macaques by IFN-γ ELISpot. The macaques received a 1:1 mixture of the pDK-Core and pDK-Pol plasmids (2 mg total plasmid) delivered IM with electroporation on days 0, 36, and 62. T-cell responses in PBMC were evaluated on day 76.

**Figure 5:**
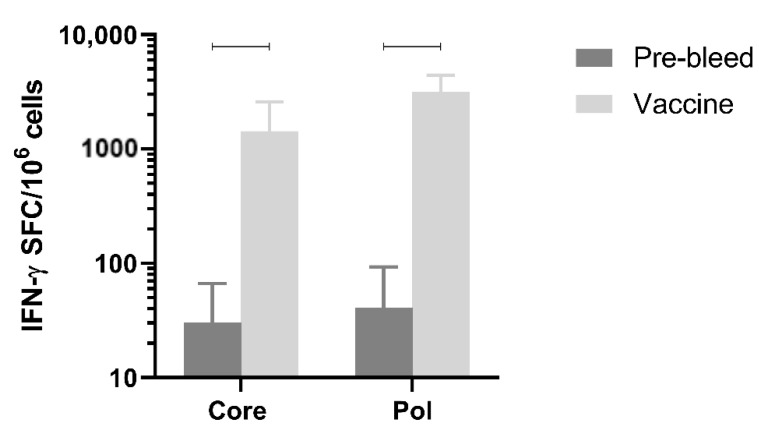
Vaccine-induced immune responses in healthy cynomolgus macaques. Core and Pol specific T-cell responses (expressed as SFC/million cells) in the PBMC of healthy cynomolgus macaques (n = 5) before (pre-bleed) and after vaccination with 1:1 mixture of pDK- Core and pDK-Pol plasmids at day 76 (dosing of 2 mg at day 0, 36 and 62). Dosing and sampling were performed as described in Materials and Methods. Statistical significance (*p* ≤ 0.05) is indicated above the bars.

The Pol response (3168.8 IFN-γ SFC/million PBMC) post-vaccination was significantly higher compared with the pre-vaccination response (41.2 IFN-γ SFC/million PBMC, *p* = 0.0005) (Figure 5). The same was observed for the vaccination-induced Core response (1436.3 IFN-γ SFC/million PBMC) compared to pre-vaccination values (30.5 IFN-γ SFC/million PBMC, *p* = 0.0021) (Figure 5). These results indicated that electroporation-mediated delivery of the DNA vaccine induced high levels of Core and Pol specific IFN-γ responses in non-human primates.

## 4. Discussion

Therapeutic vaccination might be considered a key component of a regimen for achieving functional cure in CHB patients due to the requirement for new HBV-specific T-cells capable of clearing the virus. However, to date, vaccine approaches aimed at achieving HBV functional cure have been largely unsuccessful, probably because HBsAg is not lowered [13,22,37,38]. The vaccine described herein encodes secretable proteins to enhance cross-presentation by APCs, thereby promoting generation of HBV-specific T-cells.

The major drawbacks of DNA vaccines are believed to be inefficient plasmid uptake by host cells and low levels or absence of inflammation at the injection site, resulting in suboptimal influx of antigen-presenting cells [23]. Many approaches have been utilized to improve immunogenicity, including optimization of the vaccine vectors and antigens, inclusion of molecular adjuvants, and in vivo electroporation. The latter has been shown to drive high levels of immune responses in numerous rodent and non-human primate models [24,39,40], and importantly, has been found to increase responses to DNA vaccines in multiple human clinical trials [18,19]. These previous studies showed a strong correlation between electroporation and augmented gene expression and immune responses, resulting in a significant increase in DNA vaccination efficacy. Electroporation appears not to alter the excellent safety profile of DNA vaccines or increase the risk of toxicity or integration into transfected cells, nor does it influence the spread of the plasmid to non-target tissues [41].

The original vaccine construct in this study was based on a pcDNA3.1 backbone for Core and Pol expression. The vaccine was sequence optimized, resulting in a final construct that utilizes a pVax1 backbone, with a reversed pUCori-KanR cassette containing the pUC origin of replication from pcDNA3.1 and an ampicillin promotor driving a codon-modified KanR gene. The construct is equipped with a triple enhancer and a Cystatin S signal peptide for higher protein expression and secretion, thereby increasing immunogenicity [42]. As far as we are aware, our vaccine is unique in being a DNA vaccine encoding for Core and Pol. Other HBV vaccines, including DNA vaccines, have been based on Core with HBsAg or have used adenovirus vectors [43]. Protein or peptide-based vaccines, while sometimes inducing high anti-HBV-specific antibody titers, often require continuous dosing [44]. We deliberately avoided inclusion of HBsAg in this vaccine candidate because of the possibility that HBsAg is a decoy antigen. HBsAg interacts with cell-surface heparan sulfate proteoglycans [45], potentially leading to uptake into un-infected hepatocytes, rendering them susceptible to T cell-mediated elimination [46].

Results presented here show that in both rodent and non-human primate models, the DNA vaccine induced a robust, HBV-specific immune response against Core and Pol. However, these robust responses were unable to reduce viral parameters in the AAV-HBV infection mouse model, possibly due to the high levels of immunosuppressive HBsAg [7], which might be problematic for several reasons to a therapeutic vaccine therapy. Firstly, high HBsAg levels are related to a higher frequency of myeloid-derived suppressor cells, regulatory T-cells, tumor growth factor-β, and interleukin-10 production, which all contribute to the suppression of the immune system [47]. Secondly, HBsAg also inhibits plasmacytoid dendritic cells (pDCs), reducing IFN-α production and inhibiting other functions [11], thereby reducing antiviral immunity, lowering pDC-T-cell crosstalk and pDC-induced natural killer cell function. Finally, high HBsAg levels in patients correlate with higher expression of the programmed cell death receptor, PD-1, suggesting a direct relationship to immune exhaustion [48]. Optimal efficacy of therapeutic vaccination may only be realized after first lowering HBsAg, before vaccination, thereby removing the inhibitory effects of HBsAg and allowing the full therapeutic potential of vaccine-induced T-cells [49].

Collectively, the data showed no evidence of immune interference when plasmids encoding the Core and Pol antigens were co-administered at the same site. There was no significant difference in immune responses upon administration of a mixture of Core and Pol plasmids in the same injection site compared to administering Core and Pol plasmids to different legs. Moreover, no differences in immune responses were detected when Core or Pol plasmids were applied individually.

To date, vaccine approaches for clearing CHB have largely been unsuccessful for three primary reasons. First, vaccines have induced inadequate T-cell responses [50]. Second, high HBsAg levels render the new T cells non-functional even if their induction is robust, as observed in the current study with the DNA vaccine and with other therapeutic vaccines that have been evaluated in preclinical models [49]. Finally, the patient population in clinical trials likely also has an effect on the success of a vaccine. Most vaccines are tested in patients who have already progressed (i.e., need NAs) and who tend to be older, which may limit their ability to mount effective immune responses. Vaccines may work better in the younger CHB population. For example, NASVAC (also known as ABX-203) is a combination of recombinant HBsAg and HBcAg proteins that is administered intranasally and subcutaneously. Interestingly, although no effect was seen on viral parameters in preclinical models [51], in a few CHB infected individuals (median age 54 years), it induced functional cure after 18 months of follow-up [37].

TG1050 is one of the few therapeutic vaccines that has shown an effect on viral parameters in preclinical models. TG1050 is a non-replicative adenovirus serotype 5 that encodes a fusion protein of truncated HBV Core and a modified HBV polymerase and two selected HBsAg domains, rich in T-cell epitopes [52,53]. TG1050 induced T-cell responses in mouse splenocytes from C57BL/6 mice after one injection. These responses were slightly lower than the responses measured after one vaccination with our DNA vaccine, even at low DNA dose (2 µg). When considering that, in general, C57BL/6 mice T-cell immune responses are higher compared to those in BALB/c mice, because Th1 responses are often lower in BALB/c mice [54], it might be considered that the DNA vaccine is highly immunogenic. Nevertheless, in AAV-HBV infected mice, TG1050 showed a reduction in both HBsAg and HBV DNA after multiple vaccinations [52,53], while this was not reached with the DNA vaccine under the current experimental conditions. This might be due to the difference in vaccination schedule and/or due to the inclusion of domains from envelope protein in the vaccine and/or the difference in delivery platform (DNA vaccine versus adenoviral vector). TG1050 is currently being tested in clinical trials in CHB-infected individuals [55].

In conclusion, our novel therapeutic DNA vaccine, expressing both HBV Core and Pol proteins, induced robust HBV-specific immune responses when administered intramuscularly via electroporation to naïve, AAV-HBV infected mice and healthy non-human primates. The aim of this Core and Pol vaccine was to induce mainly a Th1, cellular response capable of clearing HBV infection. This therapeutic vaccine may need to be part of a combination therapy with agents that lower HBsAg [23,36,49,56,57] and/or reverse T-cell exhaustion [58]. This needs to be further investigated.

## Figures and Tables

**Table 1:** Overview of all plasmids tested for optimal expression and secretion of Core and Pol. The table lists the names of the plasmids used in the studies with the corresponding backbone, encoded HBV protein, and a description of key features.

Name	Description	Sequence Info
pcDNA-Core	Original construct: Core expressed from pcDNA3.1	
pcDNA-Pol	Original construct: Pol expressed from pcDNA3.1	
pcDNA-CP	Core and Pol separated by small AGAG spacer in pcDNA3.1	
pcDNA-CFAP	Core and Pol separated by F2A slippage site in pcDNA3.1	AAT01756.1
pcDNA-Core-WPRE	Woodchuck post-transcriptional regulatory element (WPRE) introduced downstream of the core cds	J04514.1
pcDNA-A1-Core	Enhancer element apolipoprotein A1 precursor inserted between CMV promoter and core cds	X01038.1
pcDNA-HTLV-1-Core	Enhancer element untranslated R-U5 domain of the human T-cell Leukemia Virus Type-1 located between CMV promoter and core cds	KM023768.1
pcDNA-NTC-Core	Triple enhancer element (HTLV-1 LTR, synthetic rabbit β-globin intron, and splicing enhancer) following CMV promoter.	Splicing enhancer V00882.1
pcDNA-CystS-Core	Signal peptide Cystatin S fused to the N-terminus of the HBV core protein	NP_001890.1
pcDNA-HC-Core	Signal peptide IgG Heavy chain fused to the N-terminus of the HBV core protein	BAA75024.1
pVax1-Core	Core expressing plasmid in pVax1 backbone	Thermo Fisher Scientific, Waltham, MA, USA
pVD-Core	pVax1-Core without kanamycin antibiotics resistance cassette	
pVK-Core	Orientation of pUCori-KanR cassette (originating from pcDNA3.1) reverted in comparison to pVax1-Core	pUCori: MN996867.1
pDK (Control Vaccine)	pVax1 with pUCori-Ampprom-KanR cassette	
pDF	pVax1 with pUCori-ampicillin cassette	
pDF-Core	pDF-Core with Triple enhancer + Cystatin S	
pDF-Pol	pDF-Pol with Triple enhancer + Cystatin S	
pDK-Core	pDK-Core with Triple enhancer + Cystatin S	
pDK-Pol	pDK-Pol with Triple enhancer + Cystatin S	

## Data Availability

The data sharing policy of Janssen Pharmaceutical Companies of Johnson & Johnson is available at https://www.janssen.com/clinical-trials/transparency accessed on 26 August 2021. As noted on this site, requests for access to the study data can be submitted through Yale Open Data Access (YODA) Project site at http://yoda.yale.edu accessed on 26 August 2021.

## Supplementary Materials

The following are available online at https://www.mdpi.com/article/10.3390/vaccines9090969/s1, Figure S1. Comparison of enhancer elements, signal peptides, and backbones in HEK293T using Western blotting, Figure S2. Comparison of Core and Pol expression for different DNA fusion plasmids in HEK293T, Figure S3. Immunogenicity of DNA vaccine in healthy C57BL/6 mice, Table S1. Signal peptide sequences with indicated predicated cleavage site.
