# Supplementary material for: A Therapeutic Hepatitis B Virus DNA Vaccine Induces Specific Immune Responses in Mice and Non-Human Primates
Source: Vaccines (Basel). 2021 Aug 29;9(9):969. doi: 10.3390/vaccines9090969 (PMC8471825; doi:10.3390/vaccines9090969)
Supplement: Supplementary file 1 [file vaccines-09-00969-s001.zip › vaccines-1256097-supplementary.pdf]

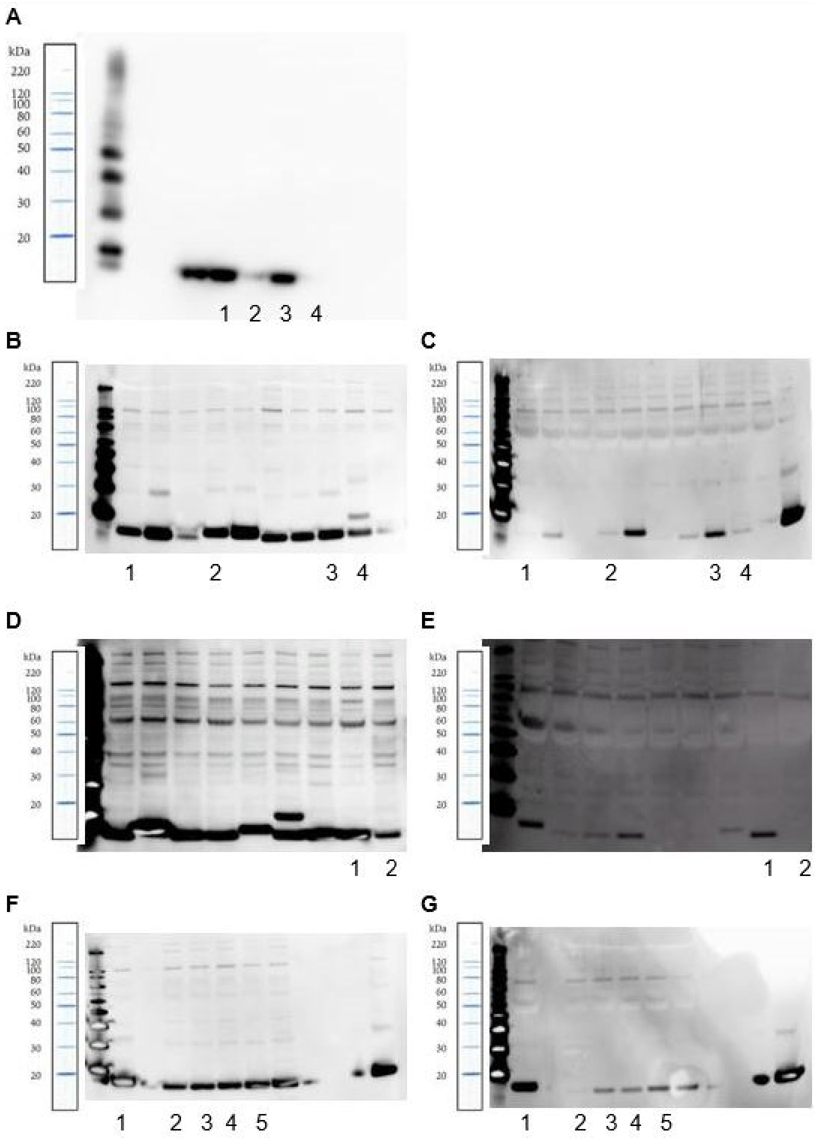

A 1 No WPRE, Lysate
2 No WPRE, Sup
3 With WPRE, Lysate
4 With WPRE, Sup

B/C 1 Control
2 A1
3 Triple
4 HTLV-1 LTR

D/E 1 Cystatin S
2 IgG Heavy Chain

F/G 1 Control D
2 V
3 VD
4 VK
5 DK

Figure S1. Western blot detection of Core protein of HEK293T cells transfected with plasmids containing different enhancer elements, signal peptides and backbones. (A) Influence of including Woodchuck Posttranscriptional Regulatory Element (WPRE) in Core expressing DNA plasmid. (B) and (C) Comparison of enhancer elements A1, triple enhancer and HTLV-1 LTR in lysate and supernatant, respectively. (D) and (E) Comparison of signal peptides cystatin S and IgG heavy chain in lysate and supernatant, respectively. (F) and (G) Comparison of backbones pcDNA3.1, pV, pVD, pVK and pDK in lysate and supernatant, respectively.

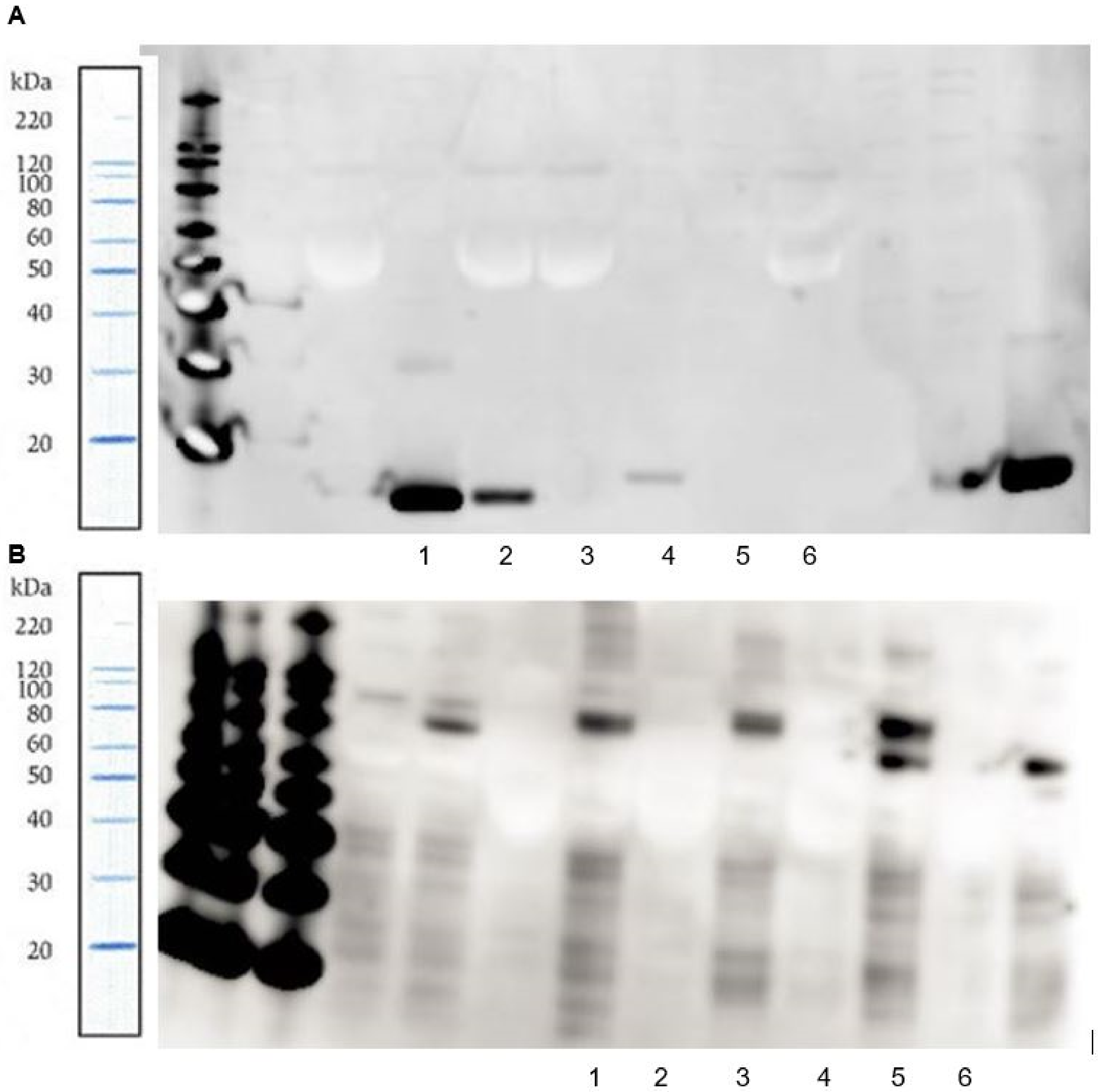

A 1 pDK-C, lysate – 2 pDK-C, sup – 3 pDK-CFAP, sup – 4 pDK-CFAP, lysate – 5 pDK-CP, lysate – 6 pDK-CP, sup

B 1 pDK-P, lysate – 2 pDK-P, sup – 3 pDK-CP, lysate – 4 pDK-CP, sup – 5 pDK-CFAP, lysate – 6 pDK-CFAP, sup

Figure S2. Western blot detection **of Core (A) and Pol (B) protein for different DNA plasmids encoding Core and/or Pol transfected in HEK293T**

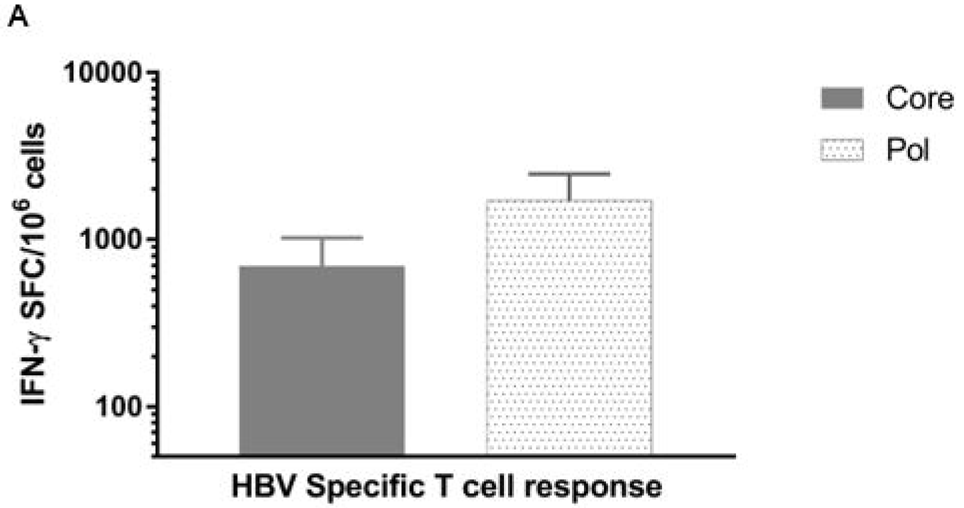

Figure S3. Immunogenicity of Core and Pol plasmids in healthy C57BL/6 mice. Core and Pol specific T-cell responses (number of IFN-γSFC)/million cells) of healthy C57BL/6 mice (n = 8 per group) vaccinated IM via electroporation with pDK-Core or pDK-Pol plasmids (10 μg per plasmid). Dosing and sampling were performed as described in the Materials and Methods.

ID.	**Sequence**	**CE**
IgG	MEFGLSWVFLVAILKGVQC-EVQLLESGM-*DID*	0.797
Cystatin S precursor	MARPLCTLLLLMATLAGALA-S*DID*	0.930

Table S1 Signal peptide sequences with indicated predicated cleavage site. Predicted cleavage sites are indicated with a hyphen. The first three amino acids of Core are indicated in italic, CE, calculated cleavage, i.e. 1.000 = 100% cleavage
